# Research and Remedies

**Published:** 1912-04-27

**Authors:** 


### RESEARCH AND REMEDIES.

### Nitro-Glycerin Poisoning.

The profound effects which all the members of the nitrite group may exert upon the circulation have long been familiar; the use of amyl nitrite for angina pectoris, and of the nitrites generally for the relief of symptoms arising from high tension, are common knowledge. Nitro-glycerin itself, the active constitutent of dynamite, has for years been officinal in the Pharmacopœia in two distinct forms. Occasional cases of poisoning from the accidental consumption of excessive doses of one or other of the nitrites have also been recorded at intervals. But although nitroglycerin is so extensively used in mining and other industrial pursuits, it does not seem hitherto to have struck anyone that it accounts for cases of severe illness or of death. Dr. Pirrie, who practises in a colliery district, believes that such cases of nitroglycerin poisoning are far from rare; and he contributes to the *Practitioner* a very full synopsis of the symptoms as they have come under his observation. He shows that many miners are accustomed to carry dynamite next to their skin for the purpose of warming it; dynamite is sufficiently pliable for easy manipulation only at a temperature of 50° F. or upwards. Men engaged in the manufacture of dynamite do not suffer from any untoward results; and Dr. Pirrie ascribes this to the fact that the work is light and that the men do not perspire over it. Miners, on the other hand, perspire freely; and the warmed, moistened dynamite may then, he thinks, be absorbed through the skin. In the series of cases reported in support of these contentions there was but one fatality; but several of the patients were for a short time seriously indisposed. Nitrites are so rapidly eliminated that it is not surprising to find the acute symptoms described as of short duration; and Dr. Pirrie's experience leads him also to believe that there is no cumulative effect. Prophylaxis, along the common-sense lines indicated by this author, is obviously the right thing for those whose susceptibility to nitrites is marked. This varying reaction to the drug is one of the chief points on which Dr. Pirrie lays stress.

### The Causes of Ascites.

A statistical investigation into the ætiology of ascites is contributed by Dr. Cabot to the *American Journal of the Medical Sciences*. This research was prompted by a series of diagnostic errors which convinced the author of the difficulty of being quite sure of the exciting factor in any given case. In all, 2,217 cases which came to autopsy were submitted to review, and some interesting conclusions were reached. In order of frequency the causes of ascites stand thus: cardiac disease, nephritis, cirrhosis of the liver, tuberculous peritonitis, intestinal obstruction, diseases of the female pelvic organs. Next to these come abdominal neoplasms and adherent pericardium. Among the cases cited, those in which the diagnosis was most difficult are naturally the most interesting. The confusion of tuberculous peritonitis with ovarian cyst, and *vice versa*, is a very easy mistake to make, and it is not surprising that there were several instances of it in the series. There were also cases of ascites due to cirrhosis of the liver in which alcoholic history and confirmatory signs were quite lacking; and nephritis also was found to be the cause of an ascites which was at first quite obscure. Another very curious case was one diagnosed as splenic anæmia with ascites; the patient was actually prepared for operation when evidence of syphilis was noted, and a course of mercury and iodides cleared up the ascites and the other symptoms.

### A New Blood-Counting Pipette.

Dr. David Thomson contributes to the *Annals of Tropical Medicine and Parasitology* a description of a new blood-counting pipette which he has invented, whereby the number of leucocytes and of blood parasites per c.m. can be estimated in one process. The method enables very much smaller droplets of blood to be used for the enumeration than when the familiar Thoma-Zeiss instrument is used; and this is of considerable advantage in dealing with laboratory animals such as mice, or with much debilitated human patients in whom frequent examinations are thought necessary. The method is simple, and seems to be about as accurate as the older plan—that is to say, the coefficient of error is not greater than 5 per cent., and can with care be reduced below this. The technique, though simple, requires a good deal of practice; and when the maximum of expertness has been attained is said to be quicker and easier than the Thoma-Zeiss technique allows. The additional advantage of being able to enumerate both white cells and blood parasites at the same sitting is another strong point in favour of Dr. Thomson's method; and it will be interesting to see whether it succeeds in ousting other instruments for these purposes in the affections of hæmatologists.

